# Efficient and Stable Proton Exchange Membrane Water Electrolysis Enabled by Stress Optimization

**DOI:** 10.1021/acscentsci.4c00037

**Published:** 2024-03-21

**Authors:** Jiawei Liu, Han Liu, Yang Yang, Yongbing Tao, Lanjun Zhao, Shuirong Li, Xiaoliang Fang, Zhiwei Lin, Huakun Wang, Hua Bing Tao, Nanfeng Zheng

**Affiliations:** † State Key Laboratory for Physical Chemistry of Solid Surfaces, Collaborative Innovation Center of Chemistry for Energy Materials, and College of Chemistry and Chemical Engineering, Xiamen University, Xiamen 361005, People’s Republic of China; ‡ Innovation Laboratory for Sciences and Technologies of Energy Materials of Fujian Province (IKKEM), Xiamen 361005, People’s Republic of China; § Fujian Key Laboratory of Digital Simulations for Coastal Civil Engineering, Xiamen University, Xiamen 361005, People’s Republic of China; ∥ College of Energy, Xiamen University, Xiamen 361005, People’s Republic of China; ⊥ Amoy Island Hydrogen (Xiamen) Technology Co. ltd, Xiamen 361101, People’s Republic of China

## Abstract

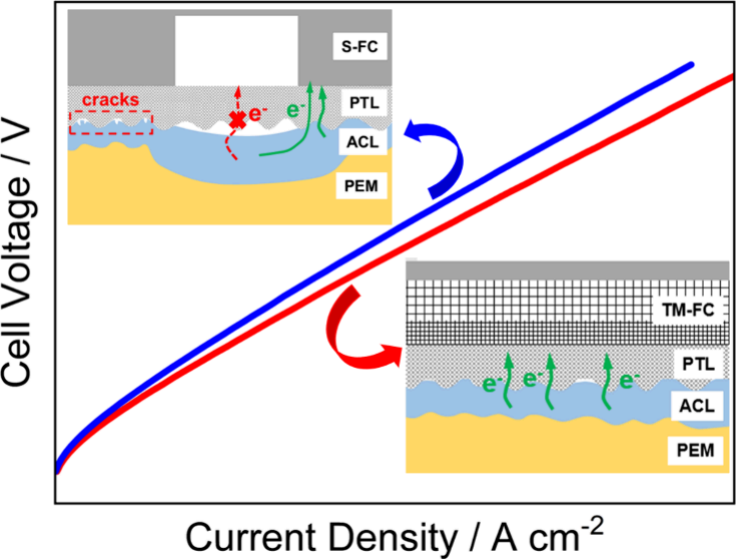

Proton exchange membrane water electrolysis (PEMWE) is a promising solution for the conversion and storage of fluctuating renewable energy sources. Although tremendously efficient materials have been developed, commercial PEMWE products still cannot fulfill industrial demands regarding efficiency and stability. In this work, we demonstrate that the stress distribution, a purely mechanical parameter in electrolyzer assembly, plays a critical role in overall efficiency and stability. The conventional cell structure, which usually adopts a serpentine flow channel (S-FC) to deliver and distribute reactants and products, resulted in highly uneven stress distribution. Consequently, the anode catalyst layer (ACL) under the high stress region was severely deformed, whereas the low stress region was not as active due to poor electrical contact. To address these issues, we proposed a Ti mesh flow channel (TM-FC) with gradient pores to reduce the stress inhomogeneity. Consequently, the ACL with TM-FC exhibited 27 mV lower voltage initially and an 8-fold reduction in voltage degradation rate compared to that with S-FC at 2.0 A/cm^2^. Additionally, the applicability of the TM-FC was demonstrated in cross-scale electrolyzers up to 100 kW, showing a voltage increase of only 20 mV (accounting for less than 2% of overall voltage) after three orders of magnitude scaleup.

## Abstract 2

A homogeneously distributed stress distribution in a gradient titanium mesh flow channel significantly enhances performance and stability of PEM water electrolysis compared to a serpentine flow channel.

## Introduction

To effectively harness the growing surplus of electricity from renewable but intermittent sources, proton exchange membrane water electrolysis (PEMWE) has emerged as a compelling solution for energy storage and green hydrogen production due to the advantages of compactness, cleanliness, and short response time.^1−4^ However, the widespread application of PEMWE is hampered by the insufficient efficiency, low durability, and high costs.^5−8^ A typical PEMWE structure consists of a porous transport layer (PTL), a flow channel (FC), catalyst layers, and a membrane.^9^ Each component is physically connected to provide efficient transport channels.^10^ Among them, the catalyst layer, where the electrochemical reaction takes place, is the most fragile component due to the combined stress of membrane creeping, uneven fibers in PTL and flow channel.^11−13^ Therefore, mechanical degradation of the catalyst layer cannot be ignored, especially under PEMWE operating conditions.

The anode side is considered the rate-determining step of water splitting due to the sluggish oxygen evolution reaction (OER). It has been reported that the areas of the anode catalyst layer (ACL) in contact with the PTL exhibited higher activity.^14−17^ Generally, the physical contact area is formed by the immersed area of PTL fibers in the ACL, which almost depends on the applied stress. Thus, the influence of different applied stresses on performance of PEMWE has been widely studied in the literature.^18−21^ The results showed that the insufficient stress results in poor contact, whereas overpressurization leads to the structural deformation of the ACL. Meanwhile, the distribution of applied stress will also greatly affect the performance of PEMWE.^22,23^ Considering the assembly process of a PEMWE device, stress is initially applied on the end plate and then transfers from the flow field plate to the internal components. Therefore, the incompressible flow field plates, fabricated with various flow channels such as parallel, serpentine, and interdigitated types, exhibit different forms of stress distribution.^24−26^ Although previous studies have reported how these flow channels affect the electron, water, and gas transport processes in PEMWE,^27−30^ none of those studies have focused on the impact of different flow channels on stress distribution of the ACL during long-term operation. Furthermore, there is a lack of research in developing an effective flow channel to optimize the PTL/ACL interface.

In this study, we investigated the effect of stress distribution on performance and durability of PEMWE. Simultaneously, we introduced a titanium mesh flow channel with a gradient pore size distribution to enhance the performance and durability of PEMWE. Our findings revealed that conventional serpentine flow channels significantly underestimated the performance and durability of ACL due to the numerous cracks induced by uneven stress applied by the channels and ridges. Conversely, the introduced titanium mesh flow channel exhibited great potential to uniformly transfer stress to the ACL/PTL interface, thereby greatly promoting in-plane electron transport in the ACL. As a result, the ACL-TM-FC showed better performance and durability than the ACL-S-FC. Moreover, the feasibility of the TM-FC was further verified from a single PEMWE cell to a PEMWE stack.

## Experimental Section

### PEMWE Design

**Figure 1:**
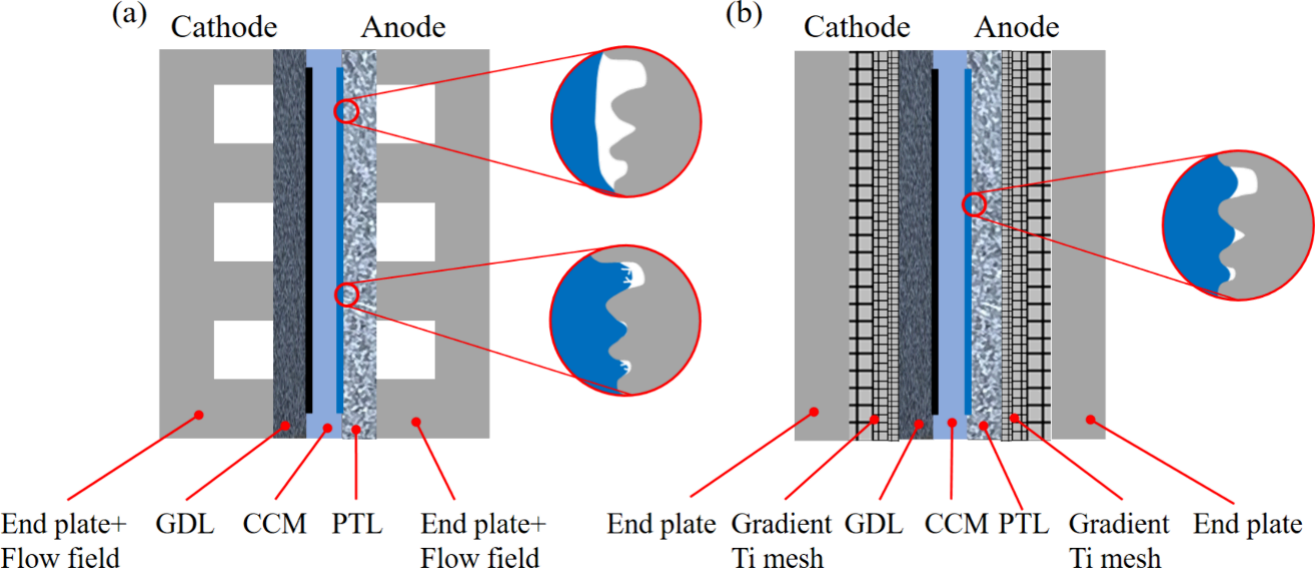
Two types of flow channel structure electrolytic cell assembly schematics: (a) S-FC; (b) TM-FC.

In this study, a self-designed PEMWE device was used. As illustrated in Figure 1a and Figure S1a, the S-FC was a single serpentine flow channel with a channel width, ridge width, and ridge height of 1 mm. And the TM-FC was a commercial Ti mesh flow channel (Zhejiang Pujiang County Changda Co., Ltd.) consisting of 5 layers of sintered titanium fibers. As shown in Figure 1b and Figure S1b, the pore sizes in the first layer are 0.15 × 0.15 mm^2^, which 1.50 × 0.75 mm^2^ in the next two layers, and 3.00 × 1.50 mm^2^ in the last two layers. The gradient structure of TM-FC is shown in Figure S1c,d.

### Catalyst-Coated-Membrane (CCM) Fabrication

The cathode ink was formulated by mixing Pt/C powder (60 wt % Pt/C, Johnson Matthey Company, UK), ionomer dispersion (D2020 from Dupont Company, USA), deionized water (18.25 MΩ cm), and isopropanol (purity ≥99.9%, from Sigma-Aldrich). The ionomer/carbon (I/C) ratio was maintained at 0.625. The anode catalyst layer comprised iridium dioxide supported on titanium dioxide (IrO_2_/TiO2_)_, Aquivion ionomers (D79), deionized water, and isopropanol, with an ionomer/catalyst mass ratio of 10/1 and a water/alcohol volume ratio of 3/2. Both cathode and anode inks were prepared through ball milling at 300 rpm for 4 h. Subsequently, the inks were uniformly coated on a PTFE substrate by using a blade coater. The catalyst layers on the substrate were dried using infrared light at 120 °C for 4 min. Following the drying process, both electrodes were transferred onto Nafion 115 membranes (127 μm, Dupont, US) or FS-990-PK membranes (90 μm, FUMATECH, Germany) through hot pressing under the conditions of 140 °C, 2 MPa, and 3 min. Catalyst loading was determined using X-ray fluorescence spectroscopy (XRF), revealing a Pt loading of 0.30 ± 0.03 mg/cm^2^ in the cathode electrodes and an Ir loading of 0.50 ± 0.05 mg/cm^2^ in the anode electrodes.

### Electrochemical Characterization

The CCM (catalyst-coated membrane) was equipped between the PTL (porous transport layer) and GDL (gas diffusion layer). Platinum-coated titanium felt (56% porosity, thickness 250 μm, Bekaert Company, Belgium) and carbon paper (TGP-H-060, 190 μm, Toray Company, Japan) were used as anode and cathode PTLs, respectively. Uncompressed PTFE was used as the gasket material. The thickness of the PTFE gasket for the S-FC matched the thickness of the PTL, and for the TM-FC, it equaled the combined thickness of the PTL and titanium mesh. The thickness of the PTFE gasket was 20% thinner than that of carbon paper. The applied torque wrench was set to 3.0 N m.

The electrolyzer testing was conducted on a four-channel testing station (LQ 4C-100, Amoy Island Hydrogen (Xiamen) Technology Co., ltd). The effective area of the PEMWE was 4.0 cm^2^, and the testing temperature was maintained at 80 °C. The anode was supplied with deionized water at a rate of 50 mL/min, while the cathode did not supply additional water. The electrical conductivity of cycled deionized water was kept below 1.0 μS/cm during the test. The performance of the electrolyzer was evaluated by using linear sweep voltammetry (LSV). The voltage scan range was set from 1.4 to 2.1 V at a scan rate of 1.0 mV/s with a 10 mV step increment (Solartron Energy Lab impedance analyzer (20 A), England). Electrochemical impedance spectroscopy (EIS) testing was conducted with a voltage step of 15 mV at frequencies ranging from 10 kHz to 100 Hz to obtain the HFR. The HFR was determined by the intersection of the Nyquist plot and the real axis. Following performance testing, potential electrochemical impedance spectroscopy (PEIS) curves were measured at 1.5 and 1.9 V with the frequency from 10 kHz to 0.1 Hz, with voltage amplitudes of 15 and 30 mV, respectively. A 10 min period was maintained at the test voltage before the PEIS measurement to ensure a stable state was achieved. An equivalent circuit model was used to analyze the EIS results.^31^ Durability testing was performed at a current density of 2.0 A/cm^2^ for about 500 h. An analysis of the cell voltage losses was conducted to better understand the variations in the different processes. The total cell voltage can be defined by the equation
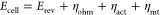
1where *E*_rev_ is the reversible cell voltage, which is approximately 1.18 V at 80 °C and 1 atm. *R*_ohm_ is the Ohmic resistance of the proton and electron transport resistance. η_HER_ and η_OER_ are the activation overpotentials of the cathode and anode, respectively. η_mt_ is the mass transport overpotential induced by water and gas flow. Because of the high activity of the platinum catalyst in the cathode, the activation loss of the hydrogen evolution reaction on the cathode side was usually ignored. The value of *R*_ohm_ is fitted by high-frequency resistance from the EIS curve, and the η_OER_ is fitted by the Tafel equation. As a result, three types of typical overpotentials can be separated.

### Scanning Electron Microscopy

The micromorphologies of ACLs were characterized by using field emission scanning electron microscopy (FE-SEM, Zeiss GeminiSEM 500, Germany). We observed the size and distribution of agglomerates on the surface and cross-section of the ACL. The cross section of the fresh and aged ACL was prepared by a Triple Ion Beam Cutter (Leica EM TIC 3X), which enables stress-free cross-section cutting and surface polishing of samples. And SEM-EDS was conducted to analyze the element content in the ACL surface before and after the durability test.

## Results and Discussion

**Figure 2:**
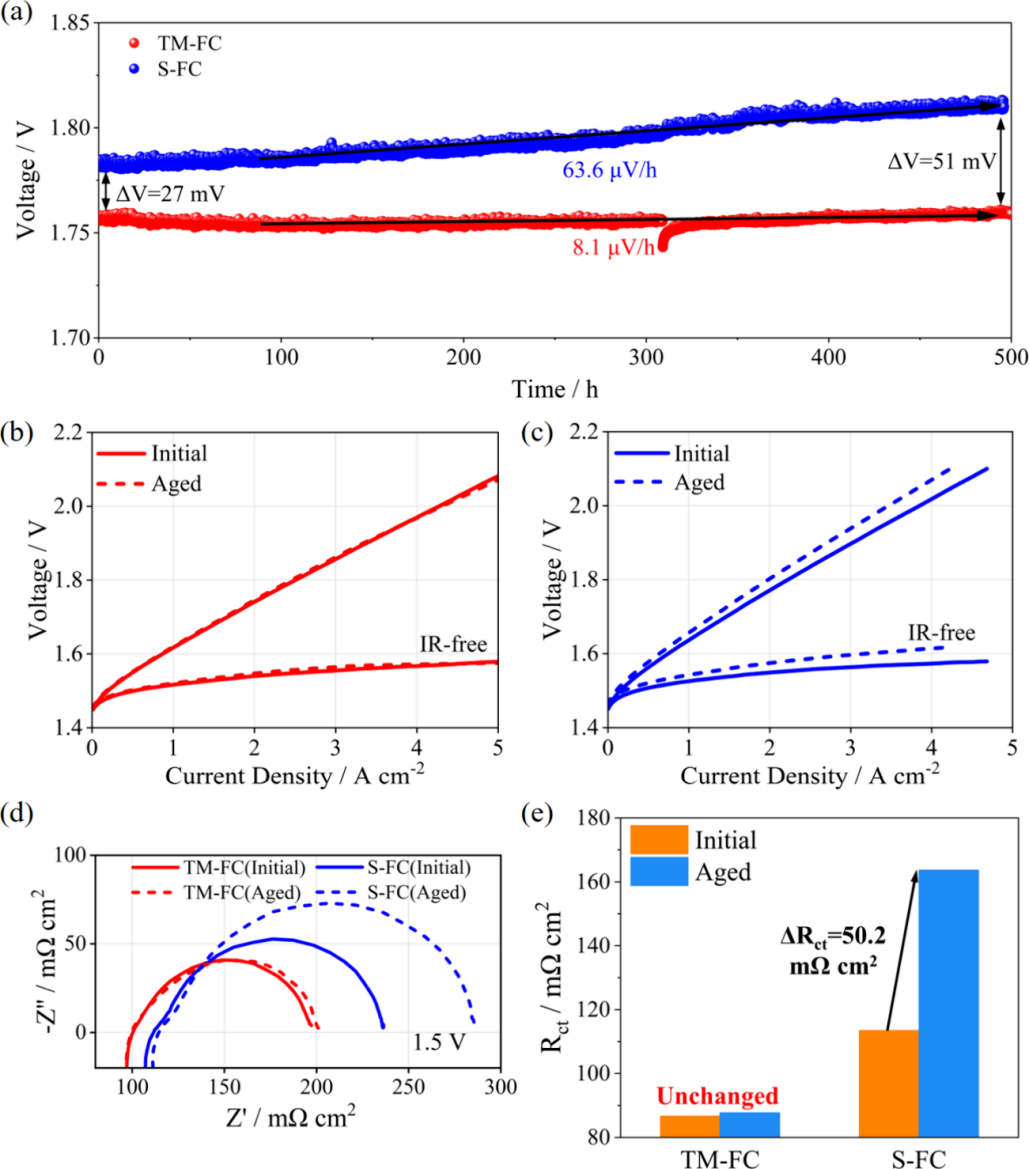
Electrochemical performance of PEMWE with ACL-TM-FC and with ACL-S-FC. (a) Durability test of ACL-TM-FC and ACL-S-FC at 2.0 A/cm^2^. (b) Polarization curve of fresh and aged ACL-TM-FC. (c) Polarization curve of fresh and aged ACL-S-FC. (d) Nyquist plots obtained at 1.5 V of fresh and aged ACL-TM-FC compared to ACL-S-FC. (e) Fitted *R*_ct_ of fresh and aged ACL-TM-FC and ACL-S-FC.

The durability of PEMWE devices was evaluated at 2.0 A/cm^2^ and 80 °C, representing relatively harsh working conditions for PEMWE. Considering the preconditioning process, the degradation rate of all ACLs was calculated after 100 h of operation. Figure 2a shows that the ACL-TM-FC had a better initial performance with a voltage reduction of about 27.0 mV compared to the ACL-S-FC. Meanwhile, the ACL-TM-FC demonstrated greater durability compared to the ACL-S-FC, with a slight degradation rate of less than 10 μV/h, while the ACL-S-FC experienced a rapid degradation rate of over 60 μV/h. Consequently, the aged ACL-TM-FC exhibited higher performance than the aged ACL-S-FC after 500 h of durability testing, with a significantly lower voltage at 2.0 A/cm^2^ (around 51.0 mV). The polarization curves shown in Figure 2b,c confirm the negligible degradation in the aged ACL-TM-FC, while revealing severe degradation in the aged ACL-S-FC. Furthermore, the loss separation illustrated in Figure S2a,b demonstrates that the degradation of the aged ACL-S-FC was caused by the increased charge transfer overpotential (η_act_) and ohmic overpotential (η_ohm_). The electrochemical impedance spectroscopy (EIS) conducted at 1.5 V as shown in Figure 2d confirmed such a degradation. The Nyquist plots were fitted using a well-established equivalent electrical circuit model.^31^ The fitted results, shown in Figure 2e and Figure S2c, indicate that the high-frequency resistance (HFR) and charge-transfer resistance (*R*_ct_) in the aged ACL-S-FC increased by 4.2 and 50.2 mΩ cm^2^, respectively. In contrast, no significant degradation was observed in aged ACL-TM-FC.

Based on above findings, we found both performance and durability were significantly improved when the ACL was equipped with TM-FC instead of S-FC. It is generally believed that optimizing the structure of the flow channel promotes the transport of water and gas, thereby improving the performance.^32,33^ However, the mass transport resistance in both FCs could be overlooked, as evidenced by the EIS curves conducted at 1.9 V (as shown in Figure S2d). Considering the reaction and transport processes in PEMWE, water splitting occurs at the triple-phase interface where electrons, protons, and water are accessible.^34^ The generated electrons transfer from the ACL to PTL, which means that the transport channels are greatly influenced by the contact area between the PTL and ACL. As ACL-TM-FC exhibited lower HFR and *R*_ct_ than the ACL-S-FC both before and after long-term running, we speculate that the enhanced performance and durability can be attributable to the optimized contact between the PTL and ACL.

To further verify our hypothesis, a detailed characterization of the microstructure of aged ACLs was conducted. Directly observing the PTL/ACL interface is challenging, because each component of the PEMWE device is physically connected during water splitting. However, the contact area between the PTL and the ACL is positively related to the force distribution. Therefore, pressure-sensitive paper serves as an effective mechanical probe to simulate the contact between PTL and ACL. As illustrated in Figure S3a,b, the pressure distribution patterns detected by pressure-sensitive paper were nearly identical to those of the flow channels, where the traces of channels and ridges were clear in ACL-S-FC (Figure S3c), while it was uniform deformation in ACL-TM-FC (Figure S3d).

**Figure 3:**
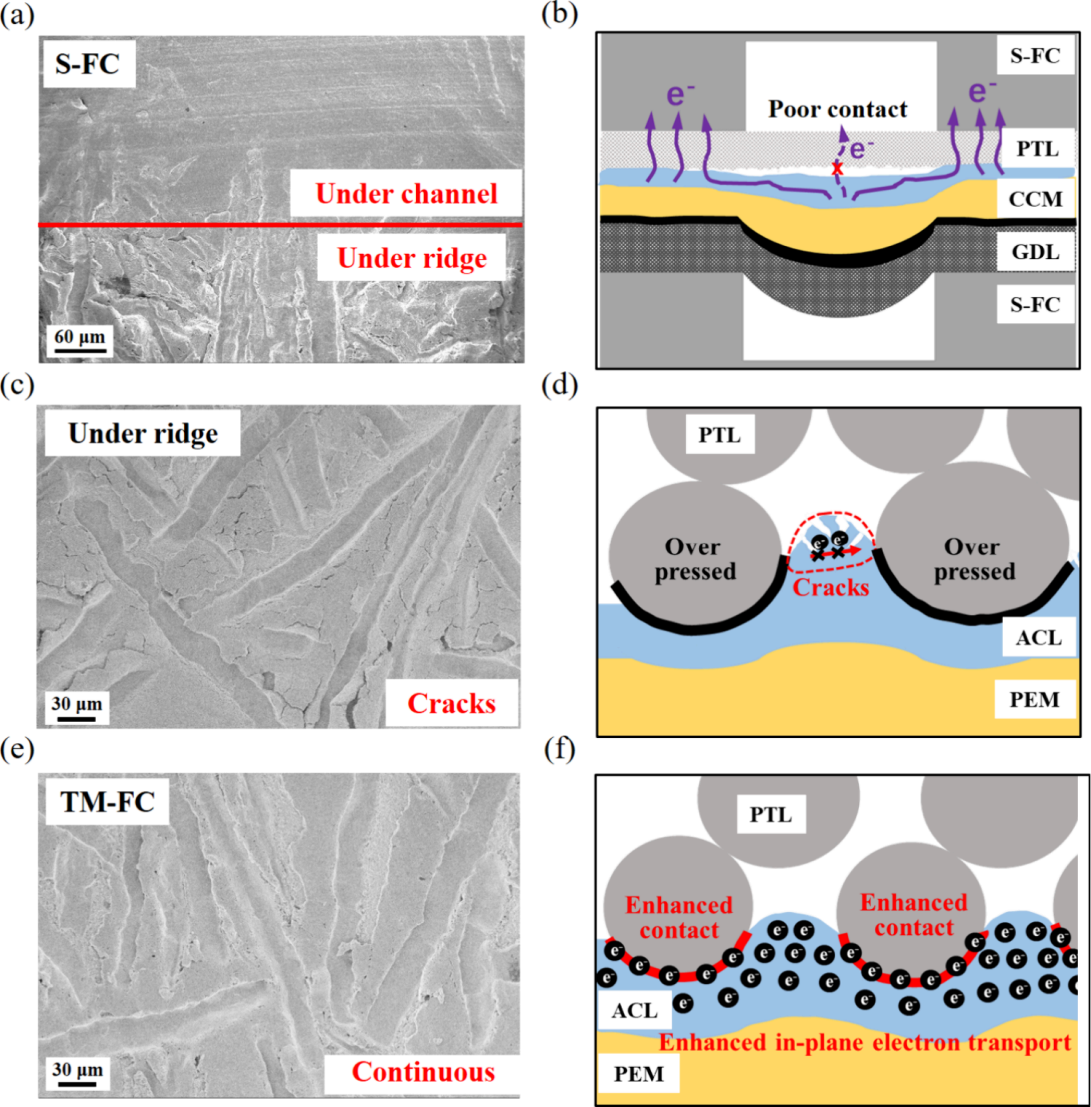
(a) SEM images of the ACL adjacent to the intersection of channel and ridge after durability testing. (b) Structural schematic of ACL under the S-FC after durability testing. (c) SEM images of ACL corresponding to the ridge of the S-FC following durability testing. (d) Structural schematic of ACL under the ridge of the S-FC after durability testing. (e) SEM images of ACL corresponding to the TM-FC following durability testing. (f) Structural schematic of ACL corresponding to the TM-FC after durability testing.

Additionally, as shown in Figure 3a, there were two distinct regions on the aged ACL-S-FC. One was a region with poor contact under the channels, and another was a connected ACL/PTL interface under the ridges. It has been reported that the areas of the ACL in contact with PTL fibers exhibited a higher activity due to the efficient electron transport channels.^35^ Consequently, as depicted in Figure 3b, the increased electron transport resistance in the area with poor contact between the ACL and PTL led to sluggish water splitting. Moreover, the region under the ridges suffered severe structural deformation, as evidenced by the numerous cracks on the aged ACL surface, which was caused by the concentration force applied by S-FC (Figure 3c). As a result, in-plane electron transport was blocked (Figure 3d), leading to an increased Ohmic resistance. However, Figure 3e,f shows that the ACL-TM-FC maintained a continuous structure due to the uniform stress distribution applied by TM-FC. Such enhanced contact between the ACL and PTL had great potential to promote in-plane electron transport. Consequently, the ACL-TM-FC had higher performance both before and after the durability test. Moreover, as presented in Movie 1a and Movie 1b, finite element simulation was conducted to reveal the different structure deformation process of CCMs with two types of flow channel structures, and the results were entirely consistent with Figure 3b,d. In S-FC, internal components were gradually deformed when applying stress. Eventually, the interface separation occurred. In contrast, the TM-FC always experienced uniform stress distribution without internal component deformation. Finally, as shown in Figure S4a,b, the structure of ACL-S-FC suffered more severe structure deformation than ACL-TM-FC due to the uneven stress distribution.

In conclusion, the performance and durability of ACL was underestimated by S-FC due to the uneven stress distribution, which resulted in severe structural deformation under PEMWE operating conditions. However, the optimized TM-FC, which had great potential to conduct uniform stress through the gradient pore distributed structure, significantly enhanced the performance and durability of ACL.

**Figure 4:**
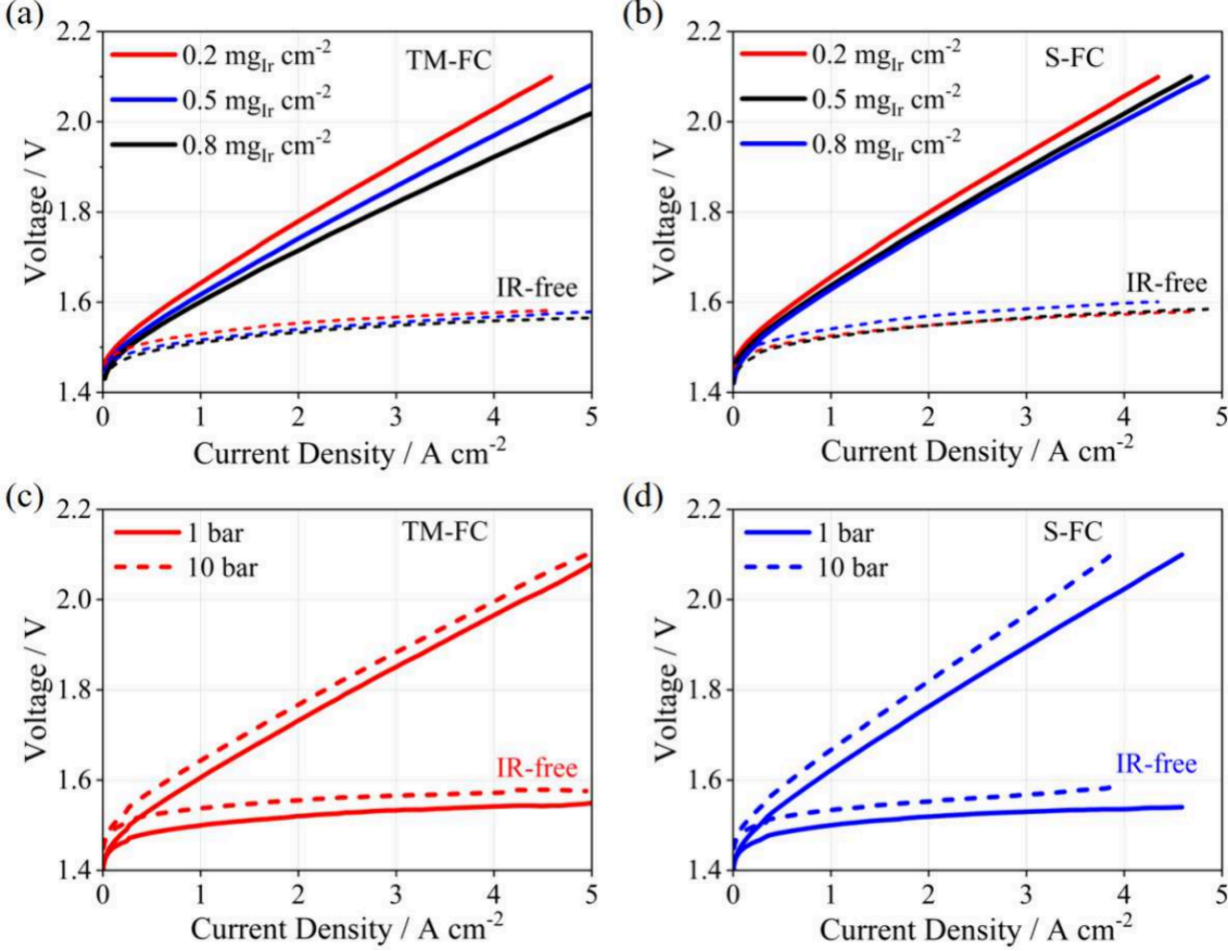
(a) Electrochemical polarization curves of ACL-TM-FC with different metal iridium loadings. (b) Electrochemical polarization curves of ACL-S-FC with different metal iridium loadings. (c) Polarization curves of ACL-TM-FC at different cathode back pressures. (d) Polarization curves of ACL-S-FC at different cathode back pressures.

To further validate the accuracy of the optimized TM-FC structure, we assessed the performance of the ACLs with varying iridium loadings. As depicted in Figure 4a,b, the performance of ACL-TM-FC exhibited a discernible gradient with different iridium loadings, whereas there was only a slight performance difference observed in ACL-S-FC. Moreover, under the same iridium loading, ACL-TM-FC consistently outperformed ACL-S-FC, as indicated by the lower cell voltage at 2.0 A/cm^2^ shown in Figure S5a. The improved performance of ACL-TM-FC, as illustrated in Figure Figure S5b–d, can be attributed to the optimized stress distribution enhancing the electron transport.

Additionally, Figure 4c,d presents the performance of both FCs under different cathode back pressure conditions. As the pressure increased, the cell voltage also increased, aligning well with the reversible cell potential derived from the Nernst equation.^36^ However, ACL-S-FC exhibited more severe degradation at 10 bar compared to ACL-TM-FC, as evidenced by the increased ohmic and mass transport resistance shown in Figure S6a,b. Similarly, Figure S6c,d demonstrates that ACL-S-FC experienced more pronounced structural deformation on both cathode and anode sides at 10 bar due to the increased inhomogeneous stress distribution at cathode high back pressure.

**Figure 5:**
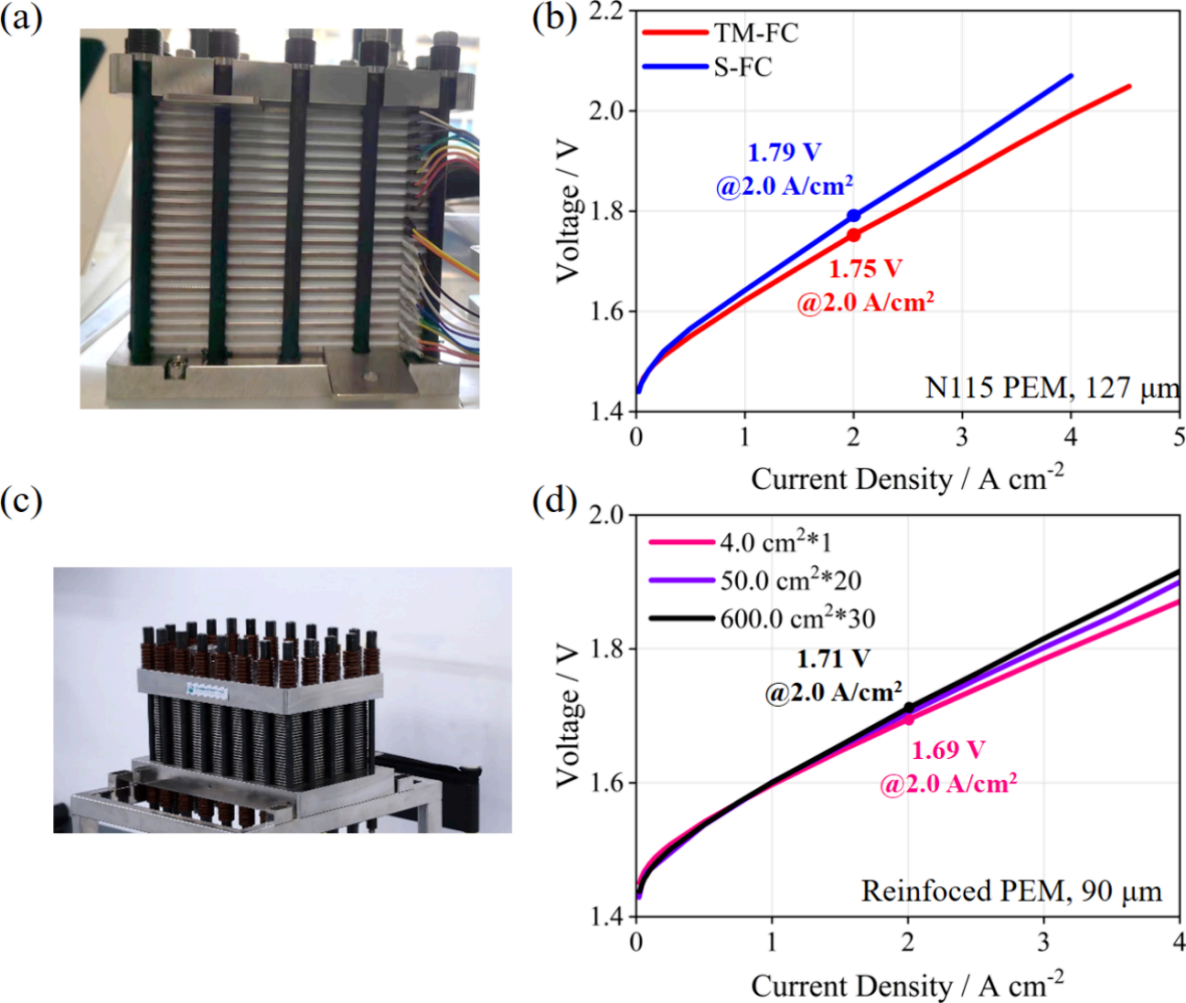
(a) Photograph of a 50.0 cm^2^ PEMWE stack. (b) Comparison of polarization curves between ACL-TM-FC and ACL-S-FC in a 50.0 cm^2^ PEMWE stack. (c) Photograph of a 600.0 cm^2^ PEMWE stack. (d) Polarization curves of ACL-TM-FC in PEMWE of different sizes.

The generalization of the TM-FC was subsequently elucidated in PEMWE devices with different sizes. Figure 5a,b presents the performance comparison between ACL-TM-FC and ACL-S-FC in a 50.0 cm^2^ PEMWE device. The results demonstrated that ACL-TM-FC consistently outperformed ACL-S-FC in this configuration. Figure 5c,d exhibits the application of TM-FC in an industrial-scale PEMWE with an active area of 600.0 cm^2^, equipped with a relatively thin membrane (90 μm, FUMASEPRFS-990-PK, Germany). Although the N115 membrane has been widely applied in the field of PEMWE, a thin membrane with high mechanical stability is expected in industrial PEMWE devices. In this work, FS-990-PK, a reinforced membrane with a thickness of 90 μm, was used to verify the reliability of the TM-FC in different sizes of PEMWE devices. It was observed that the performance of ACL-TM-FC displayed slight variations with changes in the active area. This finding further verified the reliability of TM-FC, emphasizing its consistent performance in PEMWE of different sizes.

## Conclusion

In this work, we have introduced a simple and efficient TM-FC to enhance the performance and durability of PEMWE. In comparison to the conventional S-FC, the TM-FC, featuring a gradient pore size, has demonstrated significant potential in optimizing stress distribution. Consequently, the ACL-TM-FC experienced slight structural deformation, contributing to a substantial improvement in the in-plane transport of electrons, especially during long-term operation. Furthermore, such an optimized TM-FC meets the requirements for scale-up, maintaining a high water-splitting efficiency across a broad range of active areas. Consequently, we not only present a general strategy for the reliable evaluation of PEMWE performance but also provide a promising avenue for advancements in large-scale clean energy production.

## Supporting Information Available

Additional figures as described in the text (PDF)
Finite element simulation of stress loading in S-FC (MP4)
Finite element simulation of stress loading in TM-FC(MP4)
Transparent Peer Review report available (PDF)
