# Supplementary material for: Efficient and Stable Proton Exchange Membrane Water Electrolysis Enabled by Stress Optimization
Source: ACS Cent Sci. 2024 Mar 21;10(4):852–9. doi: 10.1021/acscentsci.4c00037 (PMC11049778; doi:10.1021/acscentsci.4c00037)
Supplement: Supplementary file 1 — oc4c00037_si_001.pdf [file oc4c00037_si_001.pdf]

Supplementary Information for

Efficient and Stable Proton Exchange Membrane Water Electrolysis Enabled by Stress Optimization

Jiawei Liu[1,2#], Han Liu[1,2#], Yang Yang[1,2], Yongbing Tao[2], Lanjun Zhao[5], Shuirong Li[4], Xiaoliang Fang[4], Zhiwei Lin[1,2], Huakun Wang[3,*], Hua Bing Tao[1,2,*], and Nanfeng Zheng[1,2]

[1] State Key Laboratory for Physical Chemistry of Solid Surfaces, Collaborative Innovation Center of Chemistry for Energy Materials, and College of Chemistry and Chemical Engineering, Xiamen University, Xiamen 361005, China

[2] Innovation Laboratory for Sciences and Technologies of Energy Materials of Fujian Province (IKKEM), Xiamen 361005, China

[3] Fujian Key Laboratory of Digital Simulations for Coastal Civil Engineering, Xiamen University, Xiamen 361005, China

[4] College of Energy, Xiamen University, Xiamen 361005, China

[5] Amoy Island Hydrogen (Xiamen) Technology Co. ltd, China

*Corresponding author: hbtao@xmu.edu.cn (H. B. T.), nfzheng@xmu.edu.cn (N.Z), hkwang@xmu.edu.cn

# These authors contributed equally to this work

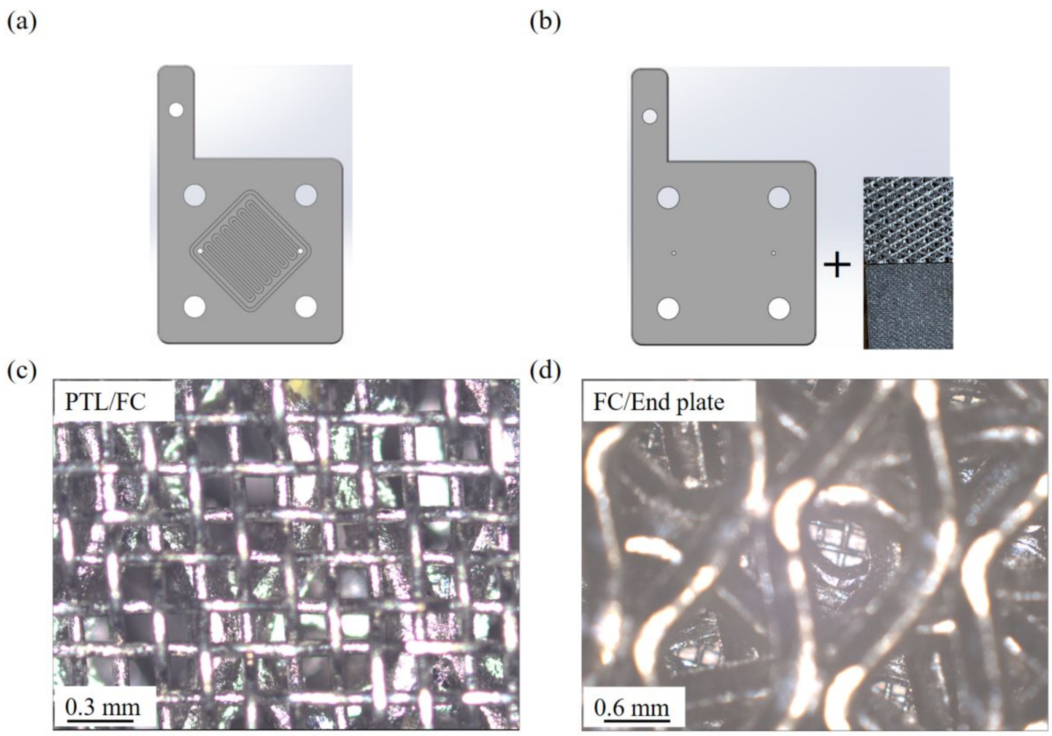

Figure S1. Flow channel structure diagram. (a) S-FC. (b) TM-FC. Morphology of TM-FC. (c) Interface between PTL and FC and (d) Interface between FC and end plate.

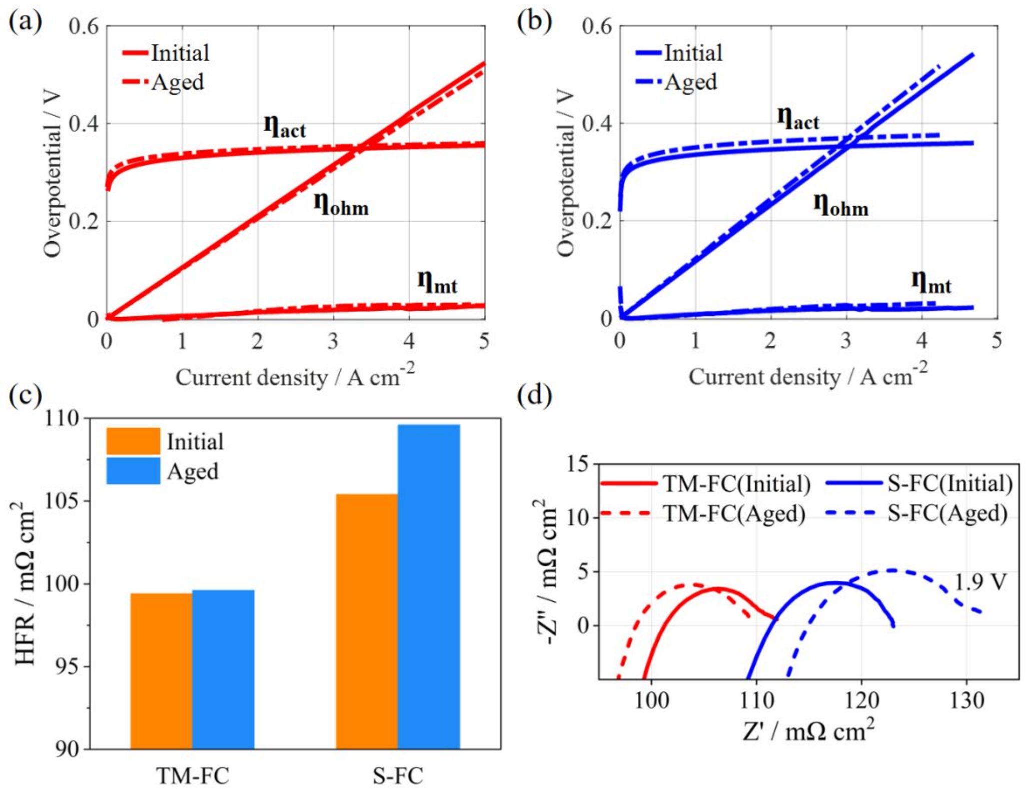

Figure S2. Study of electrochemical properties of ACL-TM-FC compared to ACL-S-FC following 500 h durability test. Loss separation of (a) TM-FC and (b) S-FC. (c) Fitted HFR at 1.5 V of TM-FC compared to S-FC. (d) Nyquist plots of PEIS conducted at 1.9 V.

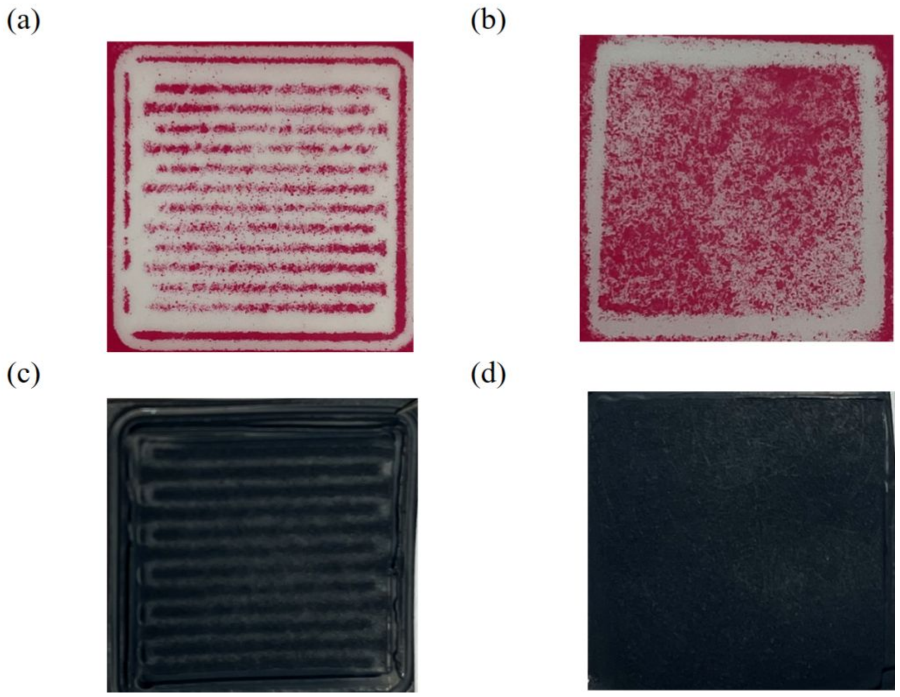

Figure S3. (a) Stress diagram at the location of the CCM in Pressure-Sensitive paper testing with S-FC. (b) Stress diagram at the location of the CCM in Pressure-Sensitive paper testing with TM-FC (c) Morphology image of the ACL after durability testing with S-FC. (d) Morphology image of the ACL after durability testing with TM-FC.

(a)

S-FC.mp4

(b)

TM-FC.mp4

Movie 1. (a) Finite element simulation of stress loading in S-FC. (b) Finite element simulation of stress loading in TM-FC.

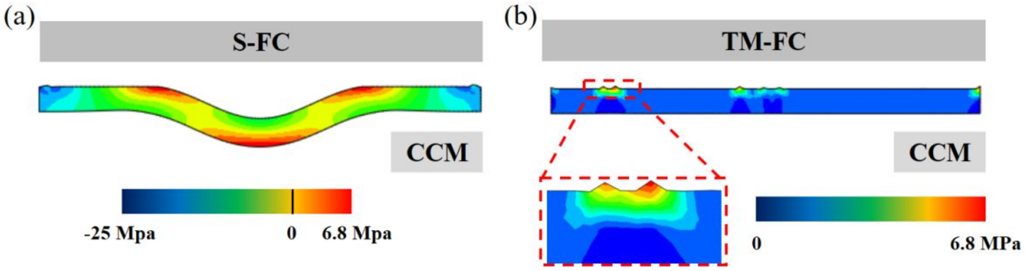

Figure S4. (a) Stress distribution contour map after stress loading in S-FC. (b) Stress distribution contour map after stress loading in TM-FC.

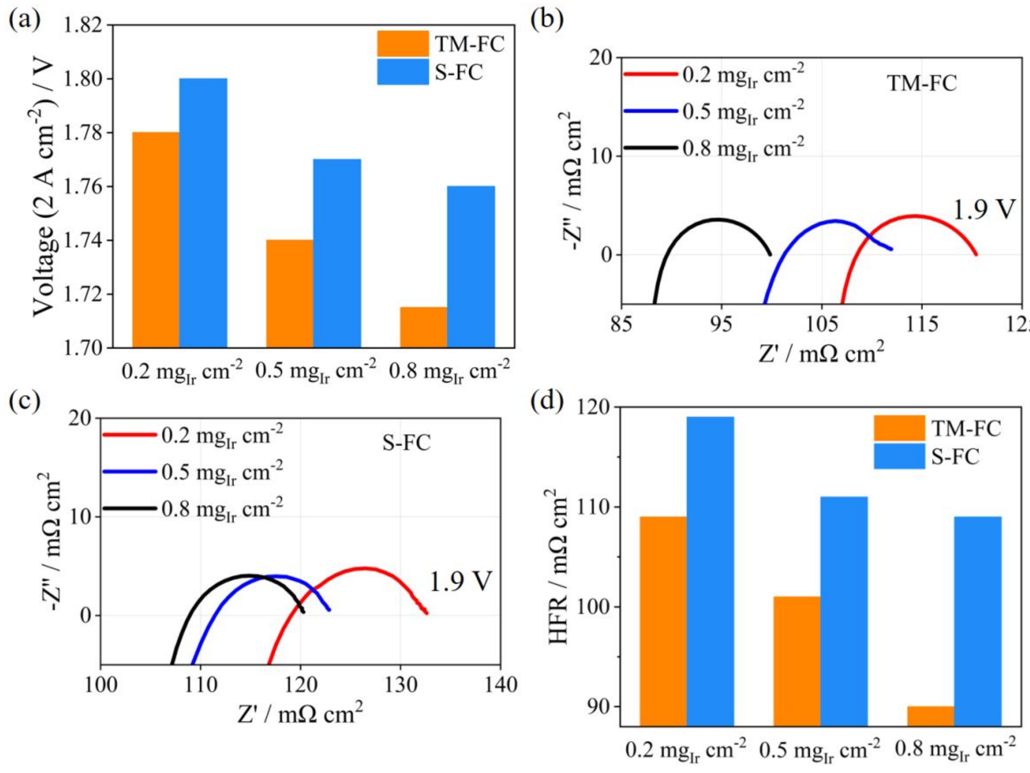

Figure S5. Testing CCM with different metal iridium loadings for two distinct flow channel structures. (a) Voltage comparison under 2 A cm^{-2}. (b) Nyquist plots of PEIS conducted at 1.9 V with ACL-TM-FC. (c) Nyquist plots of PEIS conducted at 1.9 V with ACL-S-FC. (d) Fitted HFR comparison at 1.9 V.

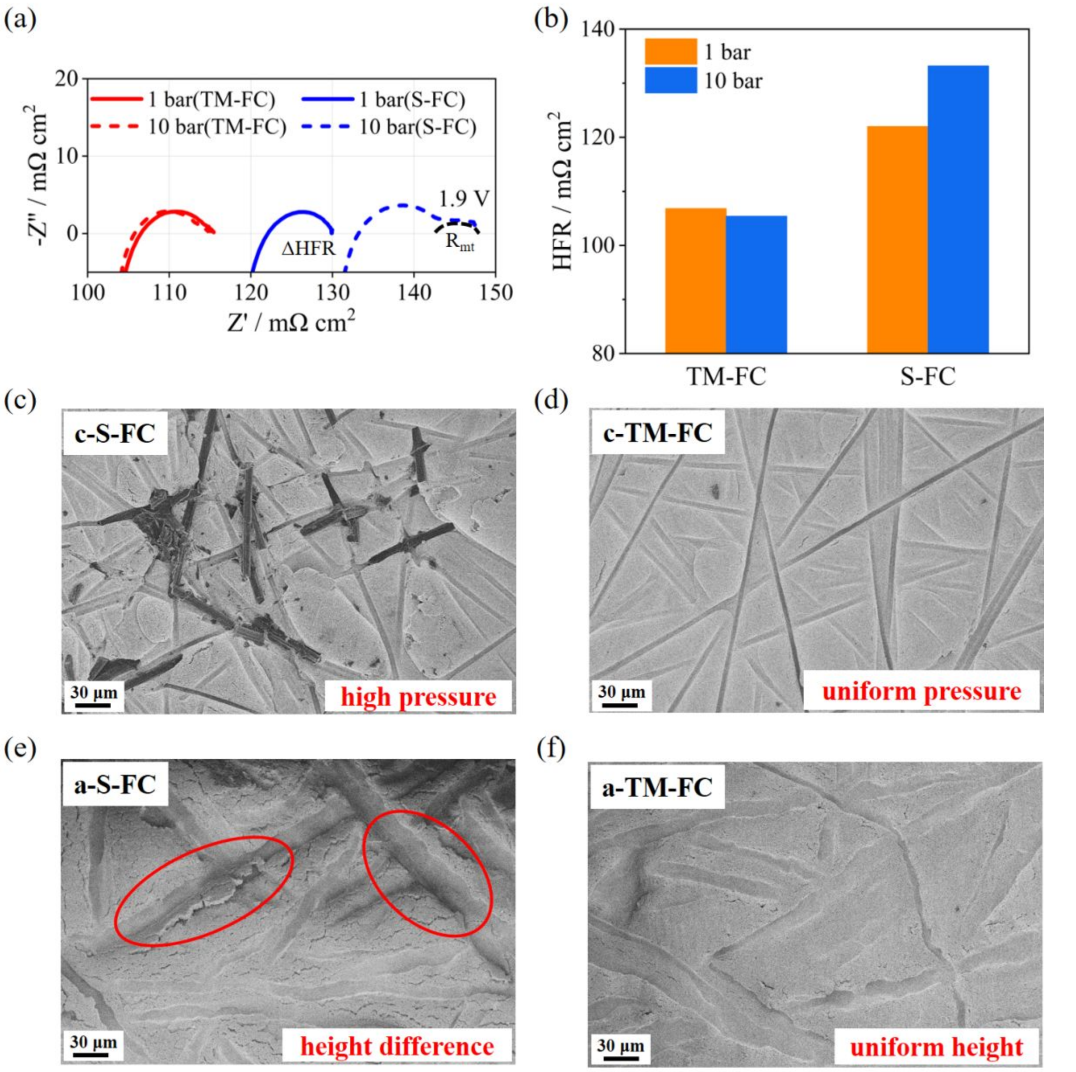

Figure S6. Testing CCM with different cathode pressure for two distinct flow channel structures. (a) Nyquist plots of PEIS conducted at 1.9 V. (b) Fitted HFR comparison at 1.9 V. (c) SEM image of cathode catalyst layer after 10 bar testing in a S-FC electrolytic cell. (d) SEM image of cathode catalyst layer after 10 bar testing in a TM-FC electrolytic cell. (e) SEM image of ACL after 10 bar testing in a S-FC electrolytic cell. (f) SEM image of ACL after 10 bar testing in a TM-FC electrolytic cell.
