# Supplementary material for: Efficient and Stable Proton Exchange Membrane Water Electrolysis Enabled by Stress Optimization
Source: ACS Cent Sci. 2024 Mar 21;10(4):852–9. doi: 10.1021/acscentsci.4c00037 (PMC11049778; doi:10.1021/acscentsci.4c00037)
Supplement: Supplementary file 4 — oc4c00037_si_004.pdf [file oc4c00037_si_004.pdf]

oc-2024-00037c.R1

Name: Peer Review Information for "Efficient and Stable Proton Exchange Membrane Water Electrolysis Enabled by Stress Optimization"

First Round of Reviewer Comments

Reviewer: 1

Comments to the Author

In this work, the authors present a study over the influence of stress on the performance of PEMWE, including efficiency and stability. Unexpectedly, the serpentine flow channel (S-FC), a most popular cell structure, proved not so good in both efficiency and stability. In contrast, a simple cell structure with gradient Ti mesh as flow channel (TM-FC) shows significantly better performance. The post-mortem characterization of catalyst coated membrane (CCM) showed that the difference was caused by the local stress distribution. Owing to the periodically ridge and channel at much larger size, the stress over CCM in S-FC is highly heterogeneously distributed, thus the catalyst layer under ridge was over pressed while that under channel was inefficiently contacted with PTL. Overall, this is an important work that bring new understanding of a cell structure and assembly parameters. It will be helpful in forming more accurate standards for fundamental cell tests. So I recommend to accept this work with a minor revision after addressing the following questions:

1) The language should be further improved;

2) The fonts size in the figure is not consistent, please keep the text consistent, in addition, the font size is too small to read (Fig.4~Fig.5, Fig.S4), The Times New Roman Fonts is recommended.

3) Fig.2 (a), for TM-FC, why did the voltage drop slightly in the first 100 hours? and what happened when the voltage dropped suddenly and rose at about 300 hours? Is it start-stop? Will it affect the subsequent performance?

4) It is a good idea to replace the S-FC with TM-FC in terms of the stress distribution, however, this may sacrifice the mass transfer performance, because the flow resistance in the tortuous porous structure increases. Will that increase the mass transfer resistance? please discuss in the paper;

5) Fig.5, why the membrane is different?

6) Fig.5, What is the design form of the flow field plate fluid distributors, will it affect the performance of electrolytic cells with different active areas?

7) The resolution of Top view and cross section view of the TM-FC should be improved in Figure S1, and the gradient characteristics of the pores should be described clearly, this is a key parameter, but it was not clearly described in the original version.

8) Movie 1 in the Supplementary Information is unable to read.

Reviewer: 2

Comments to the Author

The authors examine the effect of the stress caused by the flow channel layers to the integration and the performance of the complete PEM water electrolyzer devices and developed a simple titanium mesh flow channel that can evenly transfer the stress and enhance the overall PEMWE device performance. The work is little more on the device engineering side, but the observed stress effect is interesting. The demonstrated high performance 50 cm2 and 600 cm2 PEM electrolyzer devices are quite impressive. The manuscript can be published after the following comments are addressed:

1) It would be useful to show a image (SEM or optical, whatever is appropriate) of the the titanium mesh used here in the main text (for example in Figure 1) to give the readers a more direction idea on how does it look like and the size scale of its features.

2) I feel that there must be other more quantitative methods to represent and characterize the stress and the how uniformly the stress is distributed in both device designs, besides the images shown in Figure 3.

3) The English needs to be improved. Many of the expressions in the manuscript are quite odd. For example, just in the abstract and introduction "which demonstrated a lower cell voltage by 27 mV at"; "exhibited higher durability", "scale-up PEMWE devices"; "providing insights to achieve accurate testing"; "the titanium mesh flow channel demonstrated superior performance across different sizes of PEMWE devices," (the mesh could not demonstrate performance)... There are many more.

Author's Response to Peer Review Comments:

Dear Editor,

Thank you very much for processing our manuscript entitled " *Efficient and Stable Proton Exchange Membrane Water Electrolysis Enabled by Stress Optimization* ". We're also grateful for the positive comments

from the reviewers. In our revised manuscript, modifications are highlighted in yellow. A point-to-point reply to the reviewer's comments and suggestions is attached.

Thank you again for your kind support!

Hua Bing Tao, on behalf of all authors

Reviewer #1:

Overall: *In this work, the authors present a study over the influence of stress on the performance of PEMWE, including efficiency and stability. Unexpectedly, the serpentine flow channel (S-FC), a most popular cell structure, proved not so good in both efficiency and stability. In contrast, a simple cell structure with gradient Ti mesh as flow channel (TM-FC) shows significantly better performance. The post-mortem characterization of catalyst coated membrane (CCM) showed that the difference was caused by the local stress distribution. Owing to the periodically ridge and channel at much larger size, the stress over CCM in S-FC is highly heterogeneously distributed, thus the catalyst layer under ridge was over pressed while that under channel was inefficiently contacted with PTL. Overall, this is an important work that bring new understanding of a cell structure and assembly parameters. It will be helpful in forming more accurate standards for fundamental cell tests.* ***So I recommend to accept this work with a minor revision after addressing the following questions:***

Reponses: We thank the reviewer for the positive review of our work.

1. *The language should be further improved;*

Response: Thank you for your advice. We have revised the language expression in the revised manuscript.

2. *The fonts size in the figure is not consistent, please keep the text consistent, in addition, the font size is too small to read (Fig.4~Fig.5, Fig.S4), The Times New Roman Fonts is recommended.*

Response: Thank you for your advice. The figures and the fonts have been updated in our revised manuscript.

3. *Fig.2 (a), for TM-FC, why did the voltage drop slightly in the first 100 hours? and what happened when the voltage dropped suddenly and rose at about 300 hours? Is it start-stop?*
   Will it affect the subsequent performance?

Response: Thank you for your comments. Generally, PEMWE initially undergoes a preconditioning process, which is beneficial for removing impurities, hydrating the ionomers and building the transport channels [*ACS Appl. Mater. Interfaces* **2022**, *14* (7), 9002–9012]. Thus, the improved performance of the TM-FC assembled electrolyzer was resulted from the preconditioning process. In this work, the calculated decay rates of both ACLs were obtained after 100 hours of operation. Meanwhile, the voltage dropped and rose after 300 hours constitutes a stop-start process. Although the literature [*J. Power Sources* **2020**, *468*,

228390] have reported that the start/stop process contributed to the partial recovery of performance, which was resulted from the regeneration of the catalyst at low potentials and the escape of gas molecules trapped in the pores from the catalyst layer, the dropped voltage would quickly recovered because of establishment of a new reaction and transport equilibrium under the operating condition. Thus, a small amount of stop-start processes has negligible effect on the fitted decay rate.

4. *It is a good idea to replace the S-FC with TM-FC in terms of the stress distribution, however, this may sacrifice the mass transfer performance, because the flow resistance in the tortuous porous structure increases. Will that increase the mass transfer resistance? please discuss in the paper;*

Response: Thank you for your question. Both flow fields have negligible mass transport resistances at initial state, which is evidenced by the EIS curves conducted at 1.9 V shown in Figure 1.

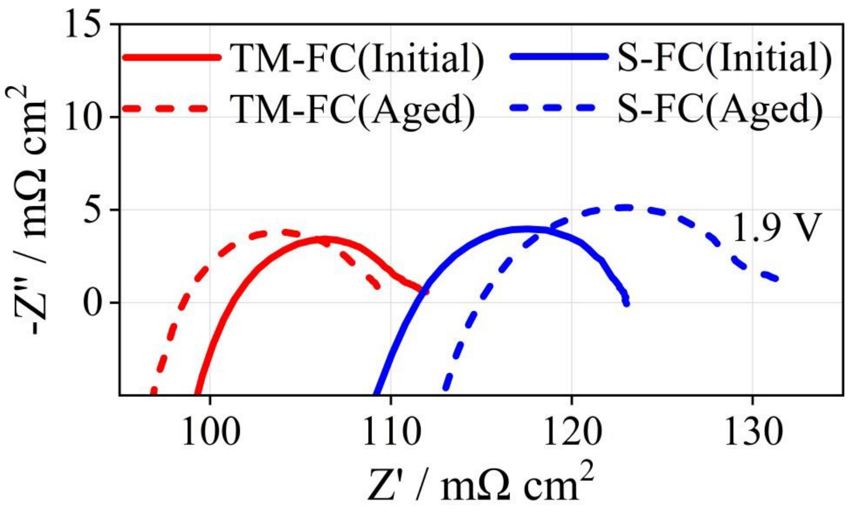

Figure 1. Nyquist plots of PEIS conducted at 1.9 V.

5. *Fig.5, why the membrane is different?*

Response: Thank you for your comments. In this study, we employed two types of proton exchange membranes, included N115 (127 μm, Dupont, US) and FS-990-PK (90 μm, FUMASEPRFS-990-PK, Germany). N115 membrane has been widely applied in the field of PEMWE. However, the thin membrane with high mechanical stability is expected in large PEMWE device. In this work, the FS-990-PK, a

reinforced membrane with a thickness of 90 μm, was used to verify the reliability of the TM-FC in different sizes of PEMWE devices.

6. *Fig.5, What is the design form of the flow field plate fluid distributors, will it affect the performance of electrolytic cells with different active areas?*

Response: Thank you for your question. In the design of the flow field plate, we primarily considered factors such as material and surface structure. Titanium metal was chosen as the base material for the flow field plate to avoid corrosion under high potentials. Additionally, we incorporated protrusions and grooves along the edges of the flow field plate to ensure a good sealing effect. The active region was mechanically polished to ensure proper contact with the TM-FC. The specific structure of the flow field plate is shown in Figure 2.

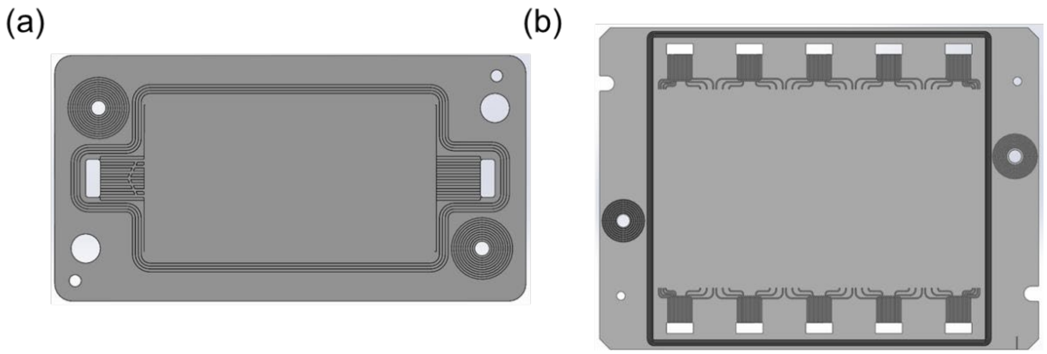

Figure 2. (a) Flow field plate structure of 50.0 cm² PEMWE. (b) Flow field plate structure diagram of 600.0 cm² PEMWE.

7. *The resolution of Top view and cross section view of the TM-FC should be improved in Figure S1, and the gradient characteristics of the pores should be described clearly, this is a key parameter, but it was not clearly described in the original version.*

Response: Thank you for your advice. The gradient structure of the Ti mesh have been added our revised manuscript, as illustrated in Figure 3. Furthermore, we have provided a detailed description of the gradient pore characteristics of the Ti mesh in the experimental section in our revised manuscript.

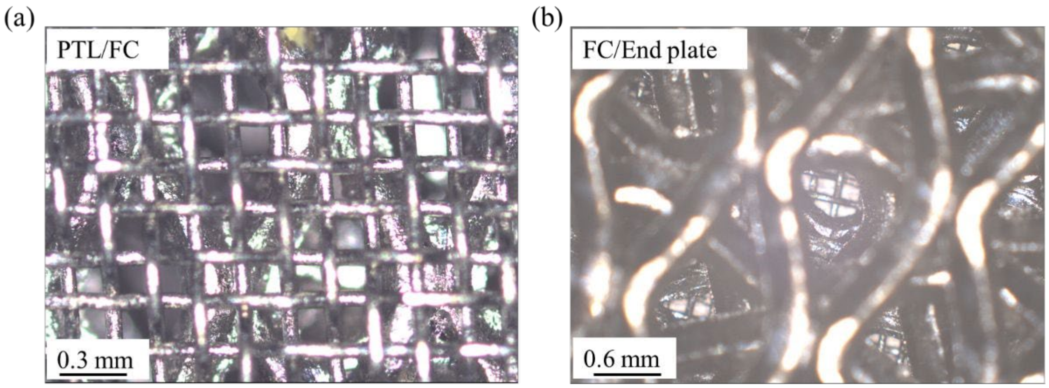

Figure 3. Morphology of TM-FC. (a) Interface between PTL and FC and (b) Interface between FC and end plate.

8. *Movie 1 in the Supplementary Information is unable to read.*

Response: Thank you for your advice. We have updated the *Movie 1* as a separated file in our revised manuscript.

Reviewer #2

Overall: The authors examine the effect of the stress caused by the flow channel layers to the integration and the performance of the complete PEM water electrolyzer devices and developed a simple titanium mesh flow channel that can evenly transfer the stress and enhance the overall PEMWE device performance. The work is little more on the device engineering side, but the observed stress effect is interesting. The demonstrated high performance 50 cm2 and 600 cm2 PEM electrolyzer devices are quite impressive. ***The manuscript can be published after the following comments are addressed:***

Reponses: We thank the reviewer for the positive review of our work.

1. *It would be useful to show a image (SEM or optical, whatever is appropriate) of the the titanium mesh used here in the main text (for example in Figure 1) to give the readers a more direction idea on how does it look like and the size scale of its features.*

Response: Thank you for your comment. As shown in Figure 1, The gradient structure of the Ti mesh have been added our revised manuscript.

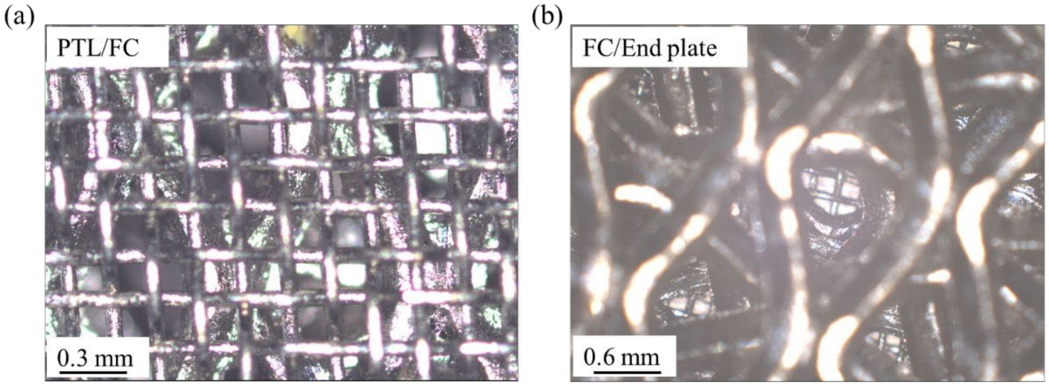

Figure 1. Morphology of TM-FC. (a) Interface between PTL and FC and (b) Interface between FC and end plate.

2. *I feel that there must be other more quantitative methods to represent and characterize the stress and the how uniformly the stress is distributed in both device designs, besides the images shown in Figure 3.*

Response: Thank you for your comments. As shown in Figure 2(a)~(d), we employed pressuresensitive paper to obtain the stress distribution inside the electrolysis cell. The test results revealed significant stress unevenness in the serpentine flow field channels and ridges, while the Ti mesh flow field notably improved the stress distribution.

Additionally, finite element analysis was employed to characterize the stress distribution inside the electrolysis cell. As presented in Figure 2(e)~(f), in a S-FC, internal components will gradually deform when applying stress. Eventually, there was a separation of interfaces. In contrast, the TM-FC experience uniform stress distribution without internal component deformation. The CCM stresses in the S-FC exhibit significant inhomogeneity, with a maximum pressure difference of up to 20 MPa between the channels and ridges, whereas the stress distribution in the TM-FC is relatively homogeneous, with significant stresses only at some protruding points of the PTL fibers.

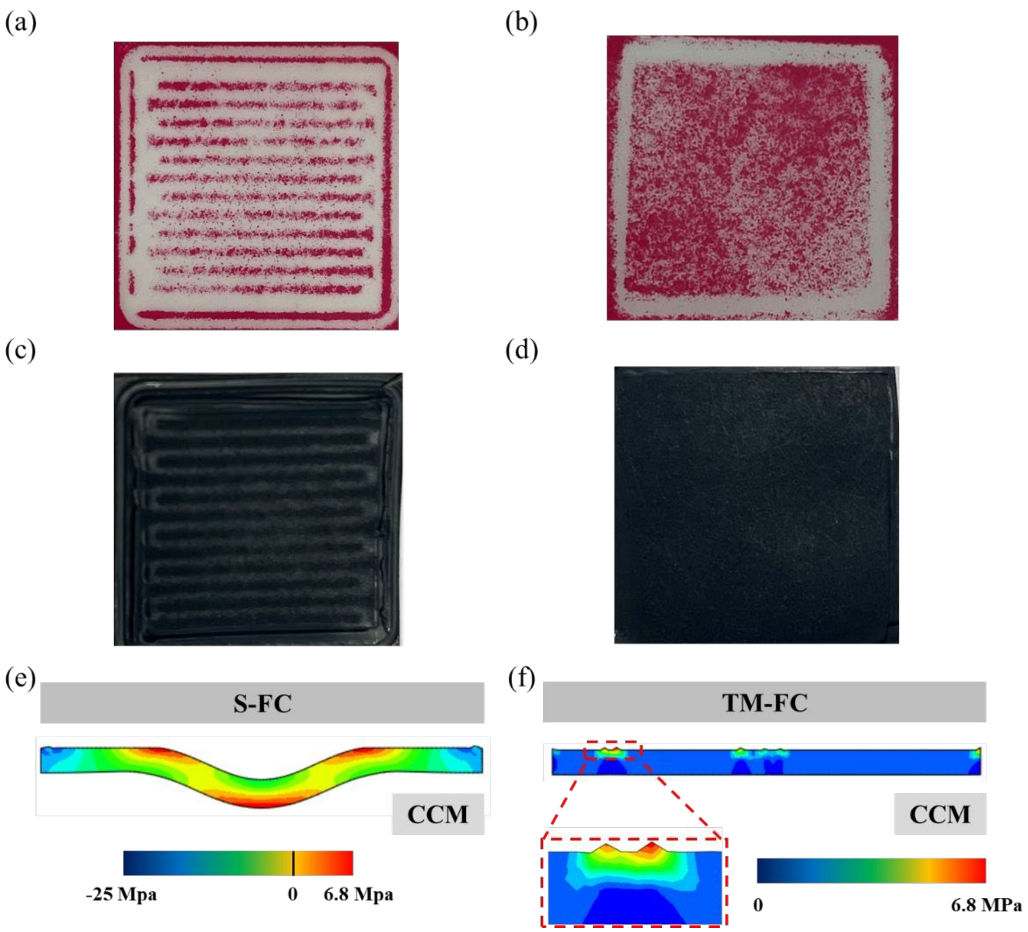

Figure 2. (a) Stress diagram at the location of the CCM in Pressure-Sensitive paper testing with S-FC. (b) Stress diagram at the location of the CCM in Pressure-Sensitive paper testing with TM-FC. (c) Morphology image of the ACL after durability testing with S-FC. (d) Morphology image of the ACL after durability testing with TM-FC. (e) Stress distribution contour map after stress loading in S-FC. (f) Stress distribution contour map after stress loading in TM-FC.

3. The English needs to be improved. Many of the expressions in the manuscript are quite odd. For example, just in the abstract and introduction "which demonstrated a lower cell voltage by 27 mV at"; "exhibited higher durability", "scale-up PEMWE devices"; "providing insights to achieve accurate testing"; "the titanium mesh flow channel demonstrated superior performance across different sizes of PEMWE devices," (the mesh could not demonstrate performance)... There are many more.

Response: Thank you for your advice. We have revised the language expression in the revised manuscript.
